# Structural data of DNA binding and molecular docking studies of dihydropyrimidinone transition metal complexes

**DOI:** 10.1016/j.dib.2018.04.040

**Published:** 2018-04-14

**Authors:** P. Vijayakrishnan, S. Arul Antony, D. Velmurugan

**Affiliations:** a Research and Development Centre, Bharathiyar University, Coimbatore 641046, Tamil Nadu, India; b PG and Research Department of Chemistry, Presidency College (Autonomous) Chennai 600005, Tamil Nadu, India; c Research Department Biophysics and Bioinformatics, University of Madras, Guindy Campus, Chennai 600025, India

**Keywords:** DHPHs, 4-aminoantipyrine, DNA binding, DNA cleavage, Antioxidant, Molecular docking

## Abstract

A series of some novel copper complexes derived from Biginelli condensation of DHPHS. The ligand and its transition metal complexes show more antimicrobial activities which was substantiated by molecular docking studies. Transition metal complexes four possess antioxidant properties supported by the DNA-binding, cleavage, and viscosity measurement (Prasad et al., 2011) [1]. The in Silicon DNA binding reveals copper complex is bound to be Minor groove and other manganese, cobalt, nickel complexes are bound to the Major groove portion of DNA through hydrogen bonds and hence copper (II), manganese (II), cobalt (II), nickel (II) complexes are called Minor groove and Major groove binder respectively. The DNA cleavage studies of metal complexes presented more protruding activity in the attendance of H_2_O_2_ associated to that in the absence of H_2_O_2._ In continuance of our ongoing research on DNA binding and cleavage happenings of transition metal complexes, in this paper we obtainable the synthesis, characterization and DNA cleavage activities.

## Graphical abstract

**Figure:**
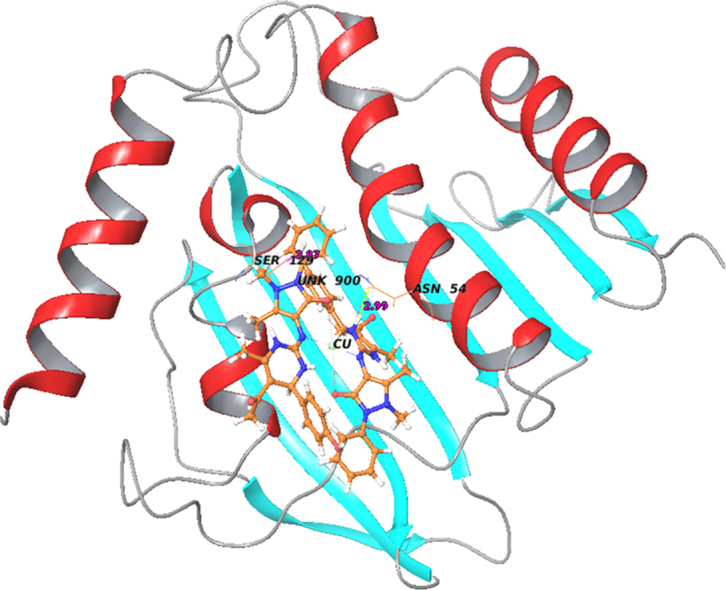

**Specifications table**

**Table:** 

Subject area	Chemistry, Biology,
More specific subject area	DNA Binding, Antioxidant, Molecular Docking
Type of data	Table, text file, graph, figure
How data was acquired	JASCO UV spectra (200–800 nm),Ubbelohde viscometer, Gel electrophoresis, superoxide dismutase, Schrodinger Maestro 9.9 OPLS-2005
Data format	Analyzed
Experimental factors	DNA binding experiments were performed in Tris–HCl/NaCl buffer (5 mmol L^−1^ Tris–HCl/50 mmol L^−^^1^ NaCl buffer pH) using DMSO (10%) solution of metal complexes. Absorption titration experiments were made using different concentration of CT-DNA, while keeping the complex concentration constant. Correction was made for the absorbance of CT-DNA.
	Viscosity experiments were conducted on the Ubbelohde viscometer, immersed in a water bath maintained at 25±0.1 °C. Titrations were performed for the compound (10–90 µl) and each compound was introduced into CT-DNA solution ( 50 µl) in the viscometer.
	DNA cleavage experiment was conducted using CT DNA by gel electrophoresis with the corresponding metal complex in the presence of H_2_O_2_ as an oxidant
	The superoxide dismutase activity (SOD) of the Mn(II), Co(II), Ni(II), Cu(II) complexes were evaluated using alkaline DMSO as source of superoxide radicals ( O_2_˙^®̅^) generating system in association with nitro blue tetrazolium (NBT) as a scavenger of superoxide, mixture were kept in ice for 15 min and then 1.5 mL of alkaline DMSO solution was added while stirring. The absorbance was monitored at 540 nm against a sample prepared under similar condition except NaOH in DMSO
	synthesized CuL_2_ and Cocrystal ligands ( 08B, NDP, 9AR, GWH, RLT) are constructed using fragment dictionary of Maestro 9.9, Totally all the docking calculation results are performed by “Extra precision” (XP) mode of Glide program
Experimental features	DNA binding studies of metal complexes ( Mn (II), Co (II), Ni (II), Cu (II)) to DNA helix has been characterized through absorption spectral titrations, significant hypochromism with a red shift of 10 nm (bathochromism) of absorption band implicates intercalative mode of binding and is likely that the all complexes with aromatic chromophore stabilizes the DNA duplex. The lower electropositive character of the metal which increases the binding mode with DNA. The electropositive character of the metal decreases as the following order: Cu(II)<Ni(II).
	Increase in viscosity of DNA as much for all M(II) complexes is observed, this increase in separation of base pairs at intercalation sites and hence an increase in DNA contour length, results from the viscosity experiments confirm the mode of these compounds intercalating into DNA base pairs.
	The double-stranded DNA tends to gradually dissociate to single strands on increase in the solution temperature and generates a hyperchromic effect on the absorption spectra of DNA bases, insertion of planar aromatic ligand in between the DNA base pairs via intercalation cause stabilization of base stack and hence raises the melting temperature of the double-stranded DNA.
	Introduction of metal group (Mn, Co, Ni, Cu) in the ligand system markedly increases the antioxidant efficient due to decreased electropositive character of metals. The activity was found in the following order MnL_2_<CoL_2_<NiL_2_<CuL_2_. Redox behaviour of the complexes responsible for its antioxidant activity, difference in reactivity of the synthesized complexes may be attributed to the coordination environment and the redox potential of the couple Cu^I^/Cu^II^ in copper(II) complex during the catalytic cycle.
	Copper complex had better glide energy and docking score compared with respect to Cocrystal ligand 3U2D-08B, 1AI9-NDP, 4CMT-GWH, 2FOM, 3OYA-RLT. Copper complex shows interaction of hydrogen bond residue and hydrophobic interaction shows with various pathogenic protein results better activity drug which are applicable through pharmaceutical and medicinal field
Data source location	Chennai, India
Data accessibility	Data is with this article

**Value of the data**

• The imine and carbonyl groups are involved in DNA binding engaged in complete insertion in between the base pairs of DNA. The strongest binding affinity exhibited by the complex is expected on the basis of aromatic ring which extend of stacking of carbonyl and imine with DNA base pairs.
• Moreover, low toxicity on the benign Vero cells and higher efficacy of the active molecules may provide a potential lead for the development of novel therapeutic agents in future.
• Transition metal complex absorption spectra of [Cu (C_24_H_25_N_5_O_3_)_2_] Cl_2_ complex in buffer pH=7.2 at 25 °C the addition of CT-DNA, that the changes in absorbance corresponding with increasing in the DNA concentration. Based on the electronic spectra, addition for DNA change to transition complex from temperature, ligand and other biological properties.
• Hydrodynamic measurements central rings of metal (M=Mn(II), Co(II), Ni(II), Cu(II) and the imine, heterocyclic keto groups are involved in intercalative mode of DNA binding, increase in viscosity of DNA is ascribed to the intercalative binding mode of the copper complexes this could cause the effective length of the DNA to increase.
• Complex shows interaction of hydrogen bond residue and hydrophobic interaction with various pathogenic protein result better activities thorough antimicrobial, anticancer, antiviral, anti-inflammatory, antigen due to. So our research demonstrates that the imines exhibit their ulcer healing properties probably due to their antioxidant action.

## 1 Data

A new series of transition metal complexes hydrodynamic measurements central rings of metal (M=Mn (II), Co(II), Ni(II), Cu(II) and imine, heterocyclic keto groups are involved in intercalative mode of DNA binding, increase in viscosity of DNA is ascribed to the intercalative binding mode of the copper complexes this could cause the effective length of the DNA to increase. The thermal behaviour of CT-DNA in the presence of complexes gave insight into their conformational changes when temperature is raised and information about the interaction strength of the complexes with DNA. The double –stranded DNA tends to gradually dissociate to single strands on increase in the solution temperature and generates a hyperchromic effect on the absorption spectra of DNA bases. DNA melting curves obtained in the presence of DNA reveal a monoplastic and irreversible melting of the DNA strands. The insertion of planar aromatic ligand in between the DNA base pairs via intercalation cause stabilization of base stack and hence raises the melting temperature of the double-stranded DNA. Metal complexes were able to convert super coiled DNA into open circular DNA. Metallo complexes bound hydroxyl radical or a peroxo species generated from the co- reactant H_2_O_2_. The DNA cleavage studies of metal complexes showed more prominent activity in the presence of H_2_O_2_ compared to that in the other metal complexes. All metal complexes showed significant nuclease activity in the presence of H_2_O_2._

## 2 Experimental design, materials and methods

The chemicals include 4-hydroxybenzaldehyde, urea, acetyl acetone, magnesium bromide, 4-aminoantipyrine, dimethylsulphoxide transition metal chlorides.

### 2.1 DNA binding studies

#### 2.1.1 Absorption titration experiments

The concentration of CT-DNA was determined from the absorption intensity at 265 nm with *ε* value of 6620 (mol L^−^^1^)^−1^. Absorption titration experiments were made using different concentration of CT-DNA, while keeping the complex concentration constant. Each sample solution was scanned from 200 to 600 nm.

#### 2.1.2 Viscosity experiments

Viscosity experiments were conducted on the Ub-belohde viscometer, immersed in a water bath maintained at 25±0.1 °C.

### 2.2 DNA cleavage studies

#### 2.2.1 Gel electrophoresis

The DNA cleavage experiment was conducted using CT DNA by gel electrophoresis with the corresponding metal complex in the presence of H_2_O_2_ as an oxidant.

### 2.3 Antioxidant assay

#### 2.3.1 Superoxide dismutase activity (SOD)

The superoxide dismutase activity (SOD) of the Mn(II), Co(II), Ni(II), Cu(II) complexes were evaluated using alkaline DMSO as source of superoxide radicals (O_2_˙^®̅^) generating system in association with nitro blue tetrazolium (NBT) as a scavenger of superoxide.

### 2.4 Molecular docking studies

#### 2.4.1 Ligand structure preparation

The synthesized CuL_2_ and Co-crystal ligands ( 08B, NDP, 9AR, GWH, RLT) are constructed using fragment dictionary of Maestro 9.9 [2] (Schrodinger, LLC) using the optimized potentials for liquid simulations of all atoms (OPLS-2005) force field with the steepest descent followed by truncated Newton conjugated protocol. Partial atomic charges are computed using the OPLS-2005 force field [3].

#### 2.4.2 Protein structure preparation

The x-ray crystal structure of proteins such as S.aureus Gyr B ATPase, Candida albicans dihydrofolate reductase, phospholipase, Human Anaplastic lymphoma kinase, Dengue Virus NS_2_B/NS_3_ protease, retroviral integrase are obtained from the Research collaborator for Bioinformatics (RCSB) Protein Data Bank are used in this study.

#### 2.4.3 Docking protocol

**Fig. 1:**
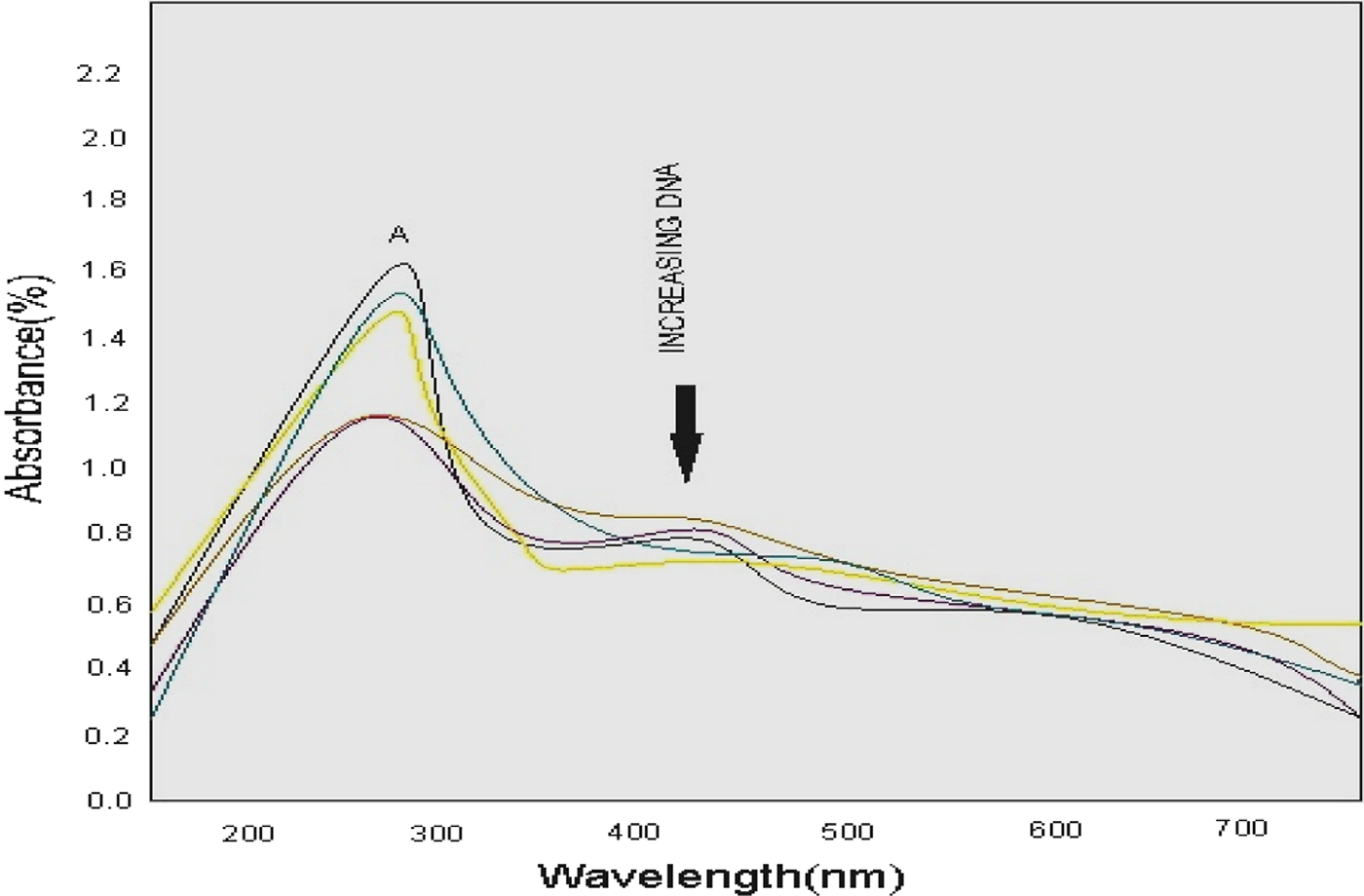
Absorption spectra of [Cu (C_24_H_25_N_5_O_3_)_2_]Cl_2_ complex in buffer pH=7.2 at 25 °C the addition of CT-DNA. Arrows indicates the changes in absorbance corresponding with increasing in the DNA concentration.

**Fig. 2:**
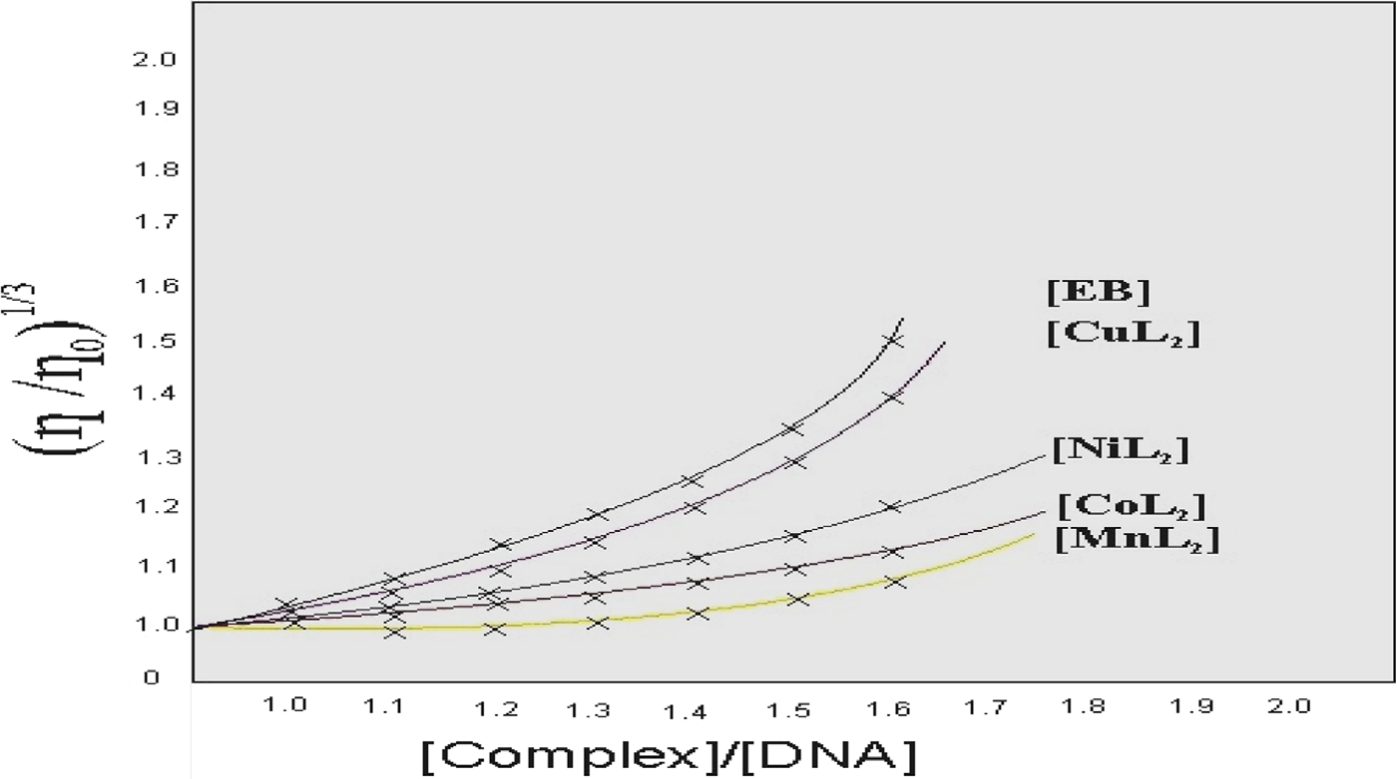
Effect on relative viscosity of CT-DNA under the influence of increasing amount of.

**Fig. 3:**
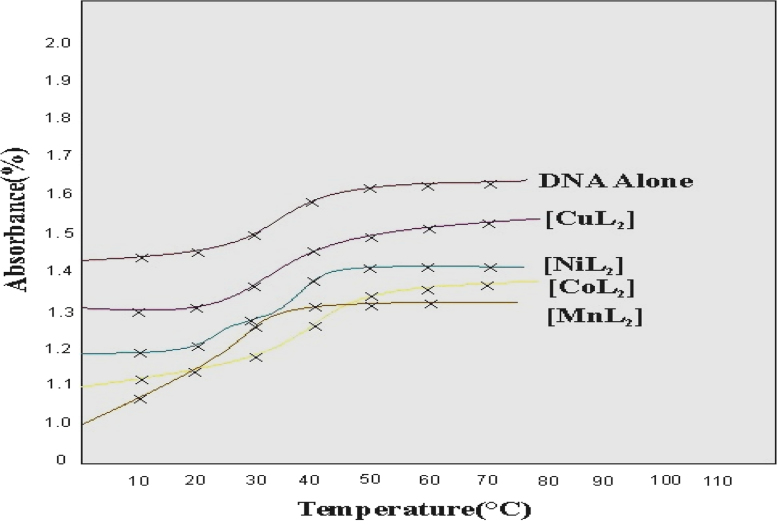
Melting curves of CT-DNA in the absence and presence of copper complex.

**Fig. 4:**
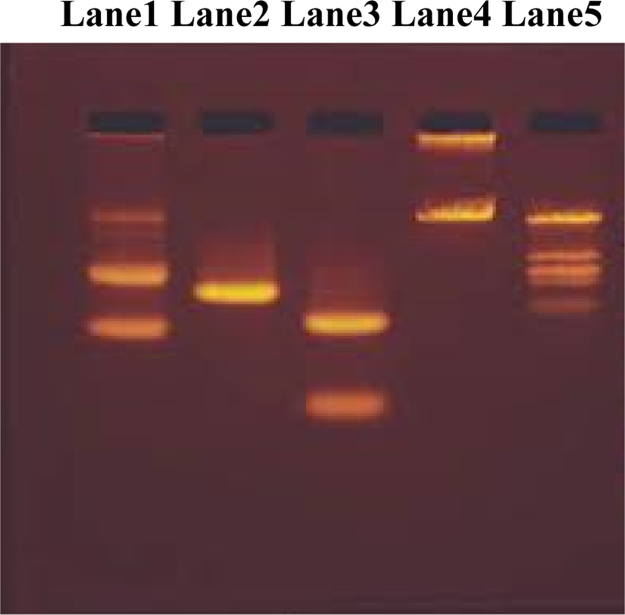
Changes in the agarose gel electrophoretic pattern of calf-thymus DNA induced by H_2_O_2_ and metal complexes: DNA alone (lane 1); DNA+[CuL_2_]Cl_2_+H_2_O_2_ (lane 2); DNA[NiL_2_]Cl_2_+H_2_O_2_ (lane 3); DNA+[CoL_2_]Cl_2_+H_2_O_2_ (lane 4); DNA+[MnL_2_]Cl_2_+H_2_O_2_ (lane 5).

**Fig. 5:**
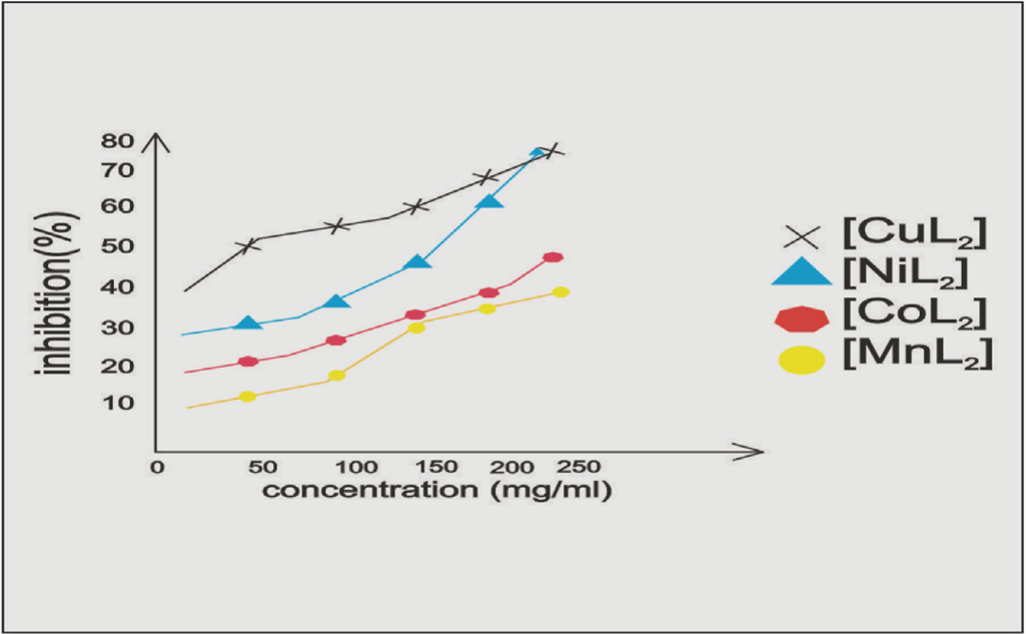
Superoxide dismutase activity of Metal(II) complexes in (µmol dm^−3^).

**Fig. 6:**
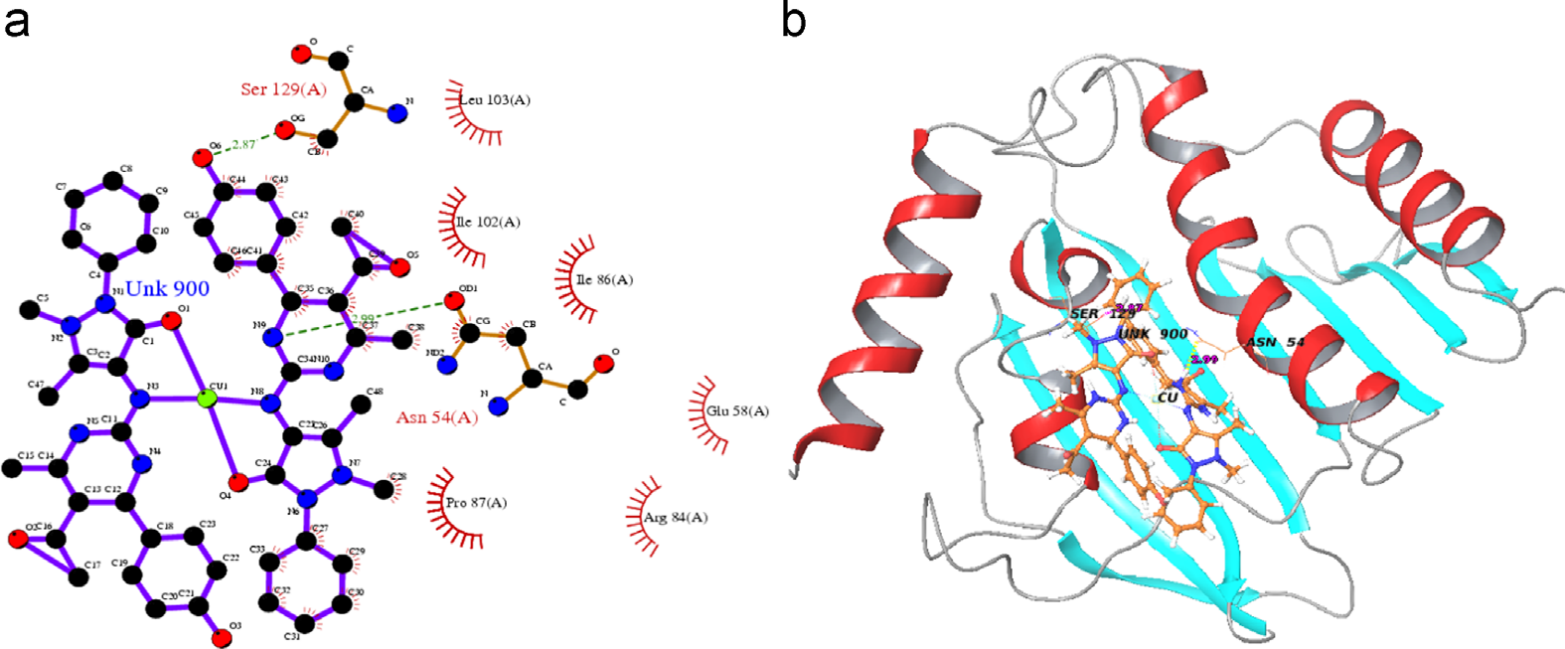
Hydrophobic and residue of hydrogen bond interaction of copper complex With *Staphylococcus aureus* bacterial protein shown in (a) ligplot(b) 3D view.

**Fig. 7:**
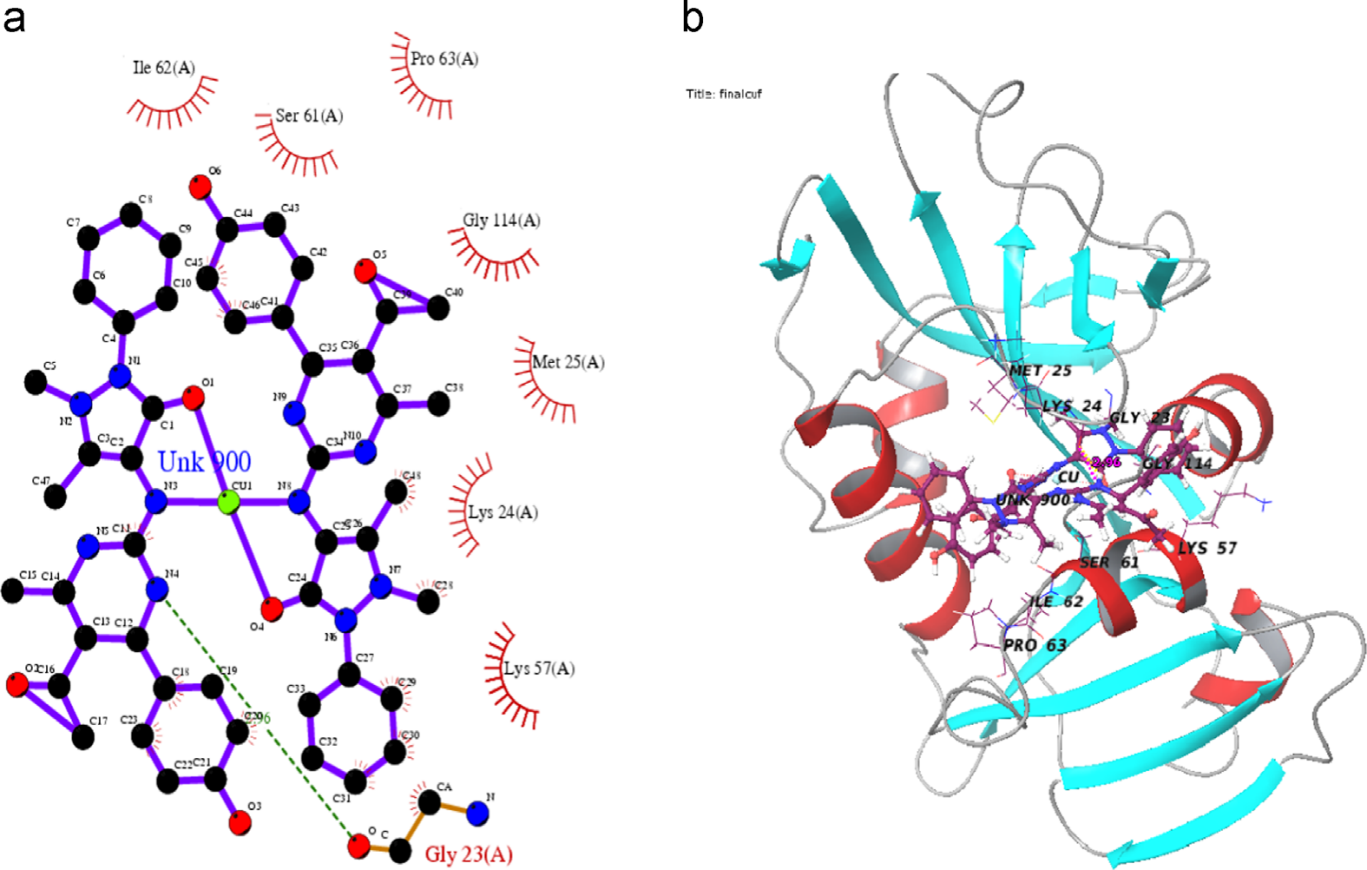
Hydrophobic and residue of hydrogen bond interaction of copper complex with *Candida albican* fungal protein (a) ligplot (b) 3D view.

Totally all the docking calculation results are performed by “Extra precision” (XP) mode of Glide program (Fig. 1, Fig. 2, Fig. 3, Fig. 4, Fig. 5, Fig. 6, Fig. 7 and Table 1, Table 2, Table 3, Table 4).

**Table 1:** Electronic spectral properties of Ni(II), Cu(II) complexes.

Compound	*λ*_max_ (nm)		Δ*λ* (nm)	a*H* (%)	*K*_b_×10^6^ (M^−^^1^)
	Free	Bound			
[NiL_2_]Cl_2_	425.3	427.5	2.2	20.0	2.3
[CuL_2_]Cl_2_	433.8	436.4	2.6	24.0	2.9

a *H*%=[*A*_free_–*A*_bound_]/[*A*_free_]×100% *K*_b=_Intrinsic DNA binding constant determined from the UV–vis absorption titration.

**Table 2:** Superoxide dismutase activity of transition metal(II) complexes.

Metal(II) complex	IC_50_ (µmol dm^−3^)
[MnL_2_]Cl_2_	40
[CoL_2_]Cl_2_	55
[NiL_2_]Cl_2_	65
[CuL_2_]Cl_2_	79

**Table 3:** Interaction of ligand and copper complex with 3U2D bacterial protein.

S. No.	Compound	Protein target	Docking score	Glide energy	Interaction of hydrogen bond residue	No. of hydrogen bonds (A)	Distance	Hydrophobic residue
ANTIBACTERIAL(3U2D)								
1	L(OH)	3U2D	−3.28	−40.213	–	–	–	Pro87(A),Arg84(A), Glu58(A),Ile86(A),
								leu103, Asn54(A),
								Thr173(A),Pro87(A)
2	CuL_2_	3U2D	−6.035	−44.199	Asn54(A)	2	2.87	Pro87(A),Arg84(A),
					Ser129(A)		2.99	Glu58(A),Ile102(A),
								Ile86(A),Leu103(A)
								Glu58(A)
3	COCRYST-08B	3U2D	−4.312	−46.747	Asp81(A)	1	2.67	Asn54(A),Gly85(A),
								Pro87(A) Ile51(A),
								Glu58(A),Ile86(A)
								Arg84(A),Thr173(A)

**Table 4:** Interaction of ligand and copper complex with 1AI9 fungal protein.

S.No.	Compound	Protein target	Docking score	Glide energy	Interaction of hydrogen bond residue	No. of hydrogen bonds (A)	Distance	Hydrophobic residue
ANTIFUNGAL(1AI9)								
1	L(OH)	1AI9	−5.487	−42.056	Ile112(A)	2	3.22	Gly114(A),Phe36(A),
					Ala115(A)		2.91	Val10(A),Ile19(A),
								Met25(A),Thr147(A),
								Lys24(A),Gly23(A),
2	CuL_2_	1AI9	−4.618	−62.173	Gly23(A)	1	2.96	Lys57(A),Lys24(A),
								Met25(A),Gly114(A),
								Pro63(A),Ser61(A),
								Ile62(A)

## References

[1] Prasad K.S., Shiva Kumar L., Chandran S., Jayalakshmi B., Revanasiddappaa H.D. (2011). Diorganotin(IV) complexes of biologically potent 4(3H)-quinazolinone derived Schiff bases: synthesis, spectroscopic characterization, DNA interaction studies and antimicrobial activity. Spectrochim. Acta Part A.

[2] Hitoshi T., Tamao N., Hideyuki A., Manabu, Takayuki M. (1997). Preparation and characterization of novel cyclic tetranuclear manganese (III) complexes: mn^III^_4_(X-salmphen)_6_ (X-salmphenH_2_=N,N′-di-substituted-salicylidene-1,3-diaminobenzene (X=H, 5-Br). Polyhedron.

[3] Punniyamurthy T., Kalra S.J.S., Iqbal J. (1995). Cobalt(II) catalyzed biomimetic oxidation of hydrocarbons in the presence of dioxygen and 2-methylpropanal. Tetrahedron Lett..

