# Supplementary material for: Structural data of DNA binding and molecular docking studies of dihydropyrimidinone transition metal complexes
Source: Data Brief. 2018 Apr 14;19:817–25. doi: 10.1016/j.dib.2018.04.040 (PMC5997584; doi:10.1016/j.dib.2018.04.040)
Supplement: Supplementary file 1 — Supplementary material [file mmc1.doc]

**Conflict of Interest Form**

**All co-authors no Conflict of Interest** We would like to submit our manuscript entitled **“ DNA binding and molecular docking studies of dihydropyrimidinone transition metal complexes"**for publication in your esteemed journal.
